# Mitigating artificial light at night: Effectiveness of policies and implications for plant phenology

**DOI:** 10.1093/pnasnexus/pgag237

**Published:** 2026-08-04

**Authors:** Brandt Geist, Lin Meng, Travis Longcore, Zhuosen Wang, Franz Hölker, Huidong Li, Hao Yin, Mahir Tajwar

**Affiliations:** Department of Earth and Environmental Sciences, Vanderbilt University, Nashville, TN 37235, USA; Department of Earth and Environmental Sciences, Vanderbilt University, Nashville, TN 37235, USA; Institute of the Environment and Sustainability, University of California, Los Angeles, Los Angeles, CA 90095, USA; Earth System Science Interdisciplinary Center, University of Maryland, College Park, MD 20742, USA; NASA Goddard Space Flight Center, Greenbelt, MD 20771, USA; Leibniz Institute of Freshwater Ecology and Inland Fisheries (IGB), Müggelseedamm 310, 12587 Berlin, Germany; Institute of Biology, Freie Universität Berlin, Königin-Luise-Straße 1-3, 14195 Berlin, Germany; Department of Earth and Environmental Sciences, Vanderbilt University, Nashville, TN 37235, USA; Department of Earth and Environmental Sciences, Vanderbilt University, Nashville, TN 37235, USA; Department of Earth and Environmental Sciences, Vanderbilt University, Nashville, TN 37235, USA

**Keywords:** artificial light at night (ALAN), plant phenology, public policy, urban ecology

## Abstract

Light pollution, an increasingly recognized environmental hazard, disrupts urban ecosystems, including plant phenology, such as leaf-out and senescence. In recent years, municipalities have enacted legislation to reduce artificial light at night (ALAN) at city levels. However, the effectiveness of these mitigation strategies on reducing ALAN and their impacts on urban ecosystems remain underexplored. Here, we present a proof of concept analysis to explore the potential of ALAN-mitigation policies on changes in intensity and spectrum composition of ALAN and consequent changes in plant phenology using Sustainable Development Science Satellite 1 data for 2022–2024 in paired US cities—one with mitigation policies and one without. Results suggest that ALAN declined in cities with ALAN-mitigation policies (New York City and Washington DC), with the largest decline in blue band. In contrast, ALAN increased in cities without policies (Newark and Philadelphia). The reduction in shifts of spring and autumn phenology observed in cities with mitigation was consistent with declines in ALAN exposure. These findings highlight the potential of multispectral nighttime light data in quantitatively assessing the efficacy of ALAN-mitigation policies in reducing light pollution and its ecological impacts.

Significance statement Artificial light at night (ALAN) is now widespread in cities and alters ecological processes, including plant seasonal cycles. This study serves as a proof of concept demonstrating how new high-resolution nighttime imagery from Sustainable Development Science Satellite 1 can be applied to assess potential effects of ALAN-mitigation efforts on light pollution levels and urban plant phenology. We observed that ALAN was reduced in cities with ALAN-mitigation policies (New York City and Washington DC) relative to their nonpolicy neighbors (Newark and Philadelphia), particularly in the blue band. These reduced ALAN was accompanied by weaker shifts in plant seasonal cycles. These findings highlight the promise of emerging satellite technologies for evaluating urban lighting strategies and their ecological relevance.

## Introduction

In recent decades, the proliferation of artificial light at night (ALAN) has become a hallmark of global urbanization, influencing ecological and social landscapes across cities worldwide (1–3). The expansion of ALAN, increasing globally at a rate exceeding 2.2% annually, correlates strongly with urban growth and is reshaping the natural nighttime environment profoundly (4, 5). This alteration in light regimes poses significant challenges to urban ecosystems, such as disrupting the natural rhythms of plants, impacting species interactions, and altering habitat suitability (6–8). Phenology, i.e. the timing of seasonal life cycle events in plants such as leaf-out, flowering, and senescence, is a critical ecological indicator and sensitive to light regime (9–11). However, the ubiquitous intrusion of ALAN in urban landscapes disrupts their light cues, leading to significant changes in plant phenological cycles, which may ripple across the ecosystem, affecting biodiversity and the functionality of ecological networks (12–14).

The impacts of ALAN on plant phenology have been found to be significant (15). Studies have shown that ALAN can advance the onset of spring events like budburst and delay autumnal processes such as leaf coloring by several days, which is comparable to the effects of climate warming on phenology (16–18). The prolonged autumnal phases under ALAN could further have cascading effects on habitat conditions and nutrient cycling (19). Additionally, the physiological stress induced by ALAN has led to metabolic disruptions in trees and serves as a growing urban stressor for both tree phenology and physiological function (20, 21). Studies have also identified specific spectral effects, such as the impact the blue light, has on accelerating spring phenological shifts, particularly in late-successional tree species (22). Field-based studies have shown that light-emitting diode (LED) streetlamps can significantly influence tree phenology in urban areas, although such effects remain understudied beyond experimental contexts (23). For these reasons, recent work has highlighted the importance of integrating ALAN into phenology models to improve ecological interpretation (24). On a broader scale, the impact of ALAN may vary with local climatic and vegetation conditions; however, the extent of this variation remains unclear (25, 26). This spatial and spectral variability suggests that managing ALAN in urban contexts may not be universally effective, necessitating tailored approaches that consider local ecological and climatic conditions (27).

Recognizing these environmental impacts, some cities have begun to implement mitigation measures aimed at reducing light pollution and preserving the natural night sky (28). For example, New York City (NYC) enacted policies that limit outdoor lighting during peak bird migration periods, mandated occupancy sensors in city buildings, and converted streetlight systems to energy-efficient LED technology (29). Washington DC's (DC) Smart Street Lighting project, initiated in spring 2022, has focused on converting 75,000 streetlights to LED technology with features that minimize blue light emissions (30). These changes could significantly modify the intensity and spectral composition of ALAN in cities, with potential implications for urban ecosystems. To refine and improve policies for different urban contexts, it is essential to assess the effectiveness of existing mitigation measures on environmental response variables like urban plant phenology, as this has rarely been done at a citywide scale (31). However, most existing nighttime light (NTL) satellite sensors, with panchromatic bands and coarse spatial resolutions, are unable to distinguish changes across the light spectrum and fine spatial-scale changes in cities that typically have high heterogeneity (5, 32). As a result, the effectiveness of these interventions in reducing ALAN and their impact on urban ecosystems remains poorly understood, creating a critical gap in urban environmental management.

The Sustainable Development Science Satellite 1 (SDGSAT-1), launched on 2021 November 29, is uniquely capable of capturing ALAN in three distinct spectral bands—red, green, and blue—at 40 m spatial resolution (32). The timely availability of this dataset allows for a first demonstration of how multispectral NTL data can be used to examine recent ALAN-mitigation policies and their potential effects on urban ecosystems in US cities. In this study, we examine the pair-city difference in relative changes in ALAN intensity, spectral composition, and subsequent changes to plant phenology in two pairs of cities: NYC and Newark, New Jersey; and DC and Philadelphia, PA, between 2022 and 2024 (Fig. S1). We hypothesize that cities implementing ALAN-mitigation measures are associated with reduced ALAN levels and consequently, reduced changes in the start of the season (SOS) and the end of the season (EOS). By comparing cities that have implemented ALAN-mitigation policies (NYC and DC) with those neighboring cities that have not (Newark and Philadelphia), we aim to demonstrate the feasibility of using SDGSAT-1 to assess the effects of these strategies on reducing ALAN levels, changing spectral composition, altering spatial variation with development levels, and the resulting impact on urban plant phenology. The findings will provide an initial proof of concept for using satellite observations from SDGSAT-1 to evaluate urban lighting solutions that protect ecological integrity while maintaining urban functionality.

## Results

### Mitigation policies are associated with reduced ALAN

**Figure 1:**
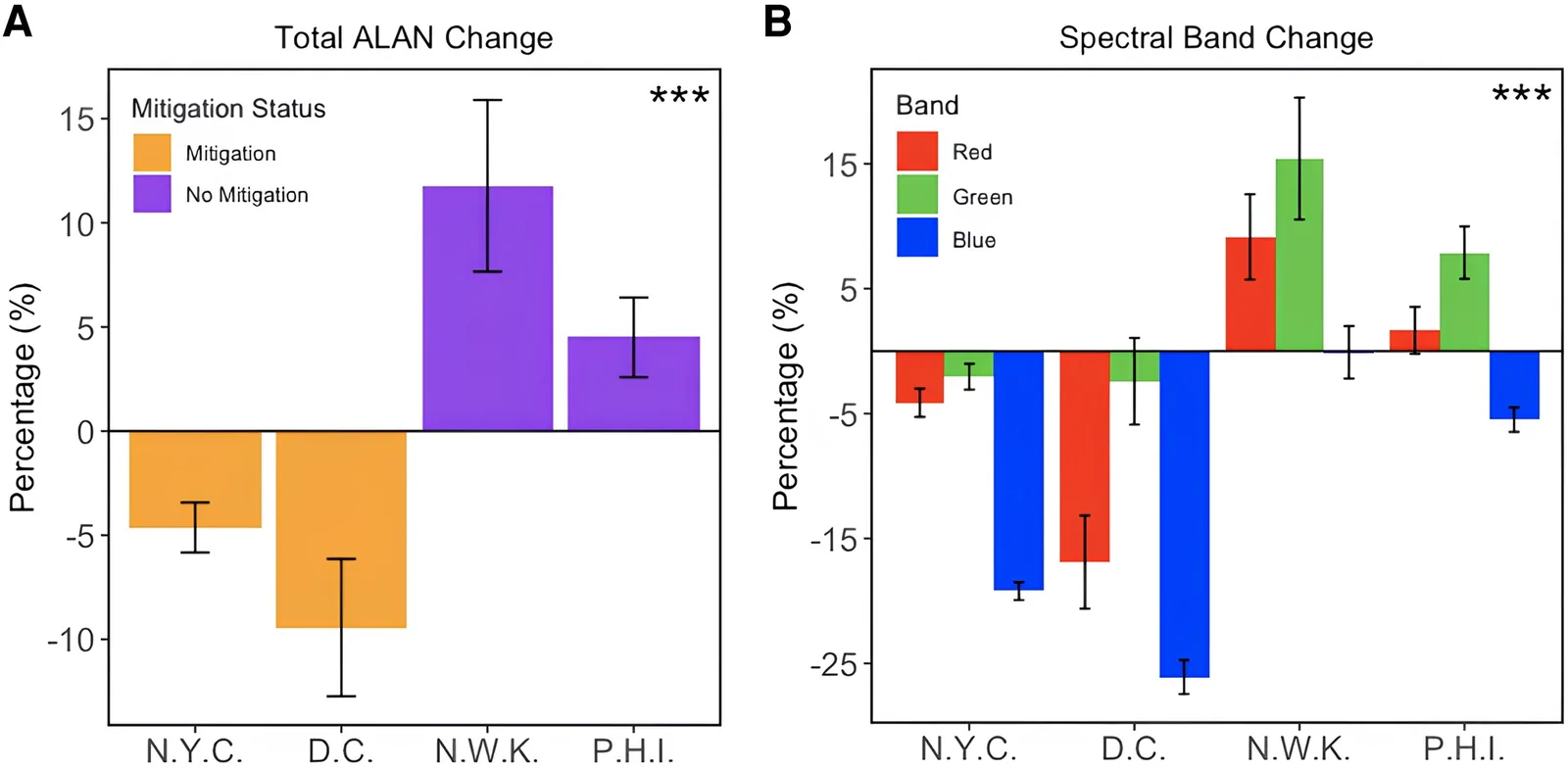
Percent change in ALAN across four study cities from 2022 to 2023. The percentage change in ALAN brightness (a) and in each spectral band (b) in each city. The percentage change was calculated by subtracting the 2022 value from the 2023 value and dividing the result by the 2022 value. N.Y.C., New York City; D.C., Washington DC; N.W.K., Newark; P.H.I., Philadelphia. Error bars represent 1 SE and *** denotes the statistically significant reductions (2023–2022) with *P* < 0.001.

Cities with ALAN-mitigation policies (NYC and DC) are associated with a decrease in NTL levels from 2022 to 2023 across spectral bands, especially in the blue (Figs. 1 and S2, Table S1). In contrast, their paired cities without policies (Newark and Philadelphia) are associated with near-zero or increasing NTL levels, with gains concentrated in the red and green bands. This temporal directional split of decreases in policy cities vs. increases in nonpolicy cities holds consistently based on total brightness (Fig. 1a) and based on each spectral band (Fig. 1b). To test the robustness of these findings, we conducted a similar analysis using a different satellite image in 2023 for NYC and Newark (Fig. S3b, Table S2), and using a newly available 2024 SDGSAT-1 satellite image for each city pair (Fig. S4c and d). Both results yielded similar ALAN change patterns across all spectral bands, reinforcing the main findings. Furthermore, we examined the ALAN changes using the Visible Infrared Imaging Radiometer Suite (VIIRS) NTL dataset for the past 5 years for DC and Philadelphia and found consistent temporal trends, i.e. decline in DC while an increase in Philadelphia (Fig. S5).

### Large spatial variation in ALAN change

**Figure 2:**
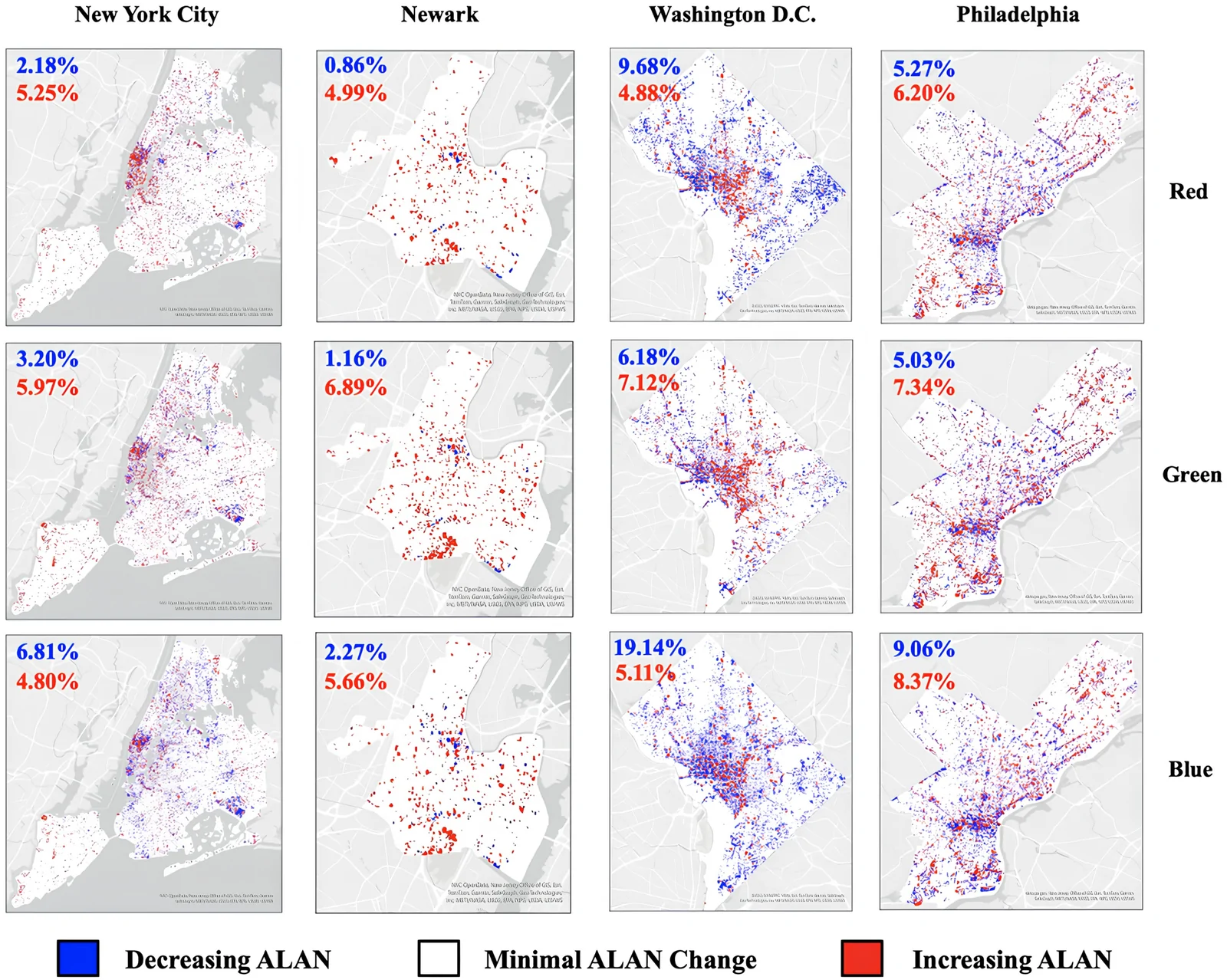
Spatial patterns of ALAN difference between 2022 and 2023 for each city and spectral band. Pixels within the range of ±mean difference of each city for each band are classified as “Minimal ALAN Change” and marked in white. Those exceeding the +mean difference are marked red (“Increasing ALAN” in 2023 compared with 2022), and those below the −mean difference are colored blue (“Decreasing ALAN”). The percentage of pixels under each classification is listed in the top left corner of each map, respectively.

The temporal changes in ALAN and its spectral composition exhibit clear spatial variation within each city (Fig. 2). In NYC, ALAN appeared to decrease in specific pockets, although the borough of Manhattan (located in the central part of the city) shows large contiguous regions with increasing ALAN. Conversely, without mitigation, Newark presents virtually no areas in the city experiencing an observed decrease in ALAN, implying a broad, citywide challenge in managing light pollution. Certain areas, like the south region near Newark Liberty International Airport, saw a notable increase in ALAN, possibly related to airport construction in 2021 and 2022, and normal usage resumed in 2023 (33).

DC exhibited an observed decrease in ALAN across a considerable portion of the city for each of the three spectral bands, with the most pronounced changes in the blue band. Furthermore, across all spectral bands, DC had a lower percentage of pixels with notable ALAN increase and a greater percentage of pixels with notable ALAN decrease compared with Philadelphia (Fig. 2). Philadelphia demonstrated a heterogeneous pattern, with both increases and decreases in ALAN across neighborhoods.

### Heterogeneous ALAN change across urban development levels

**Figure 3:**
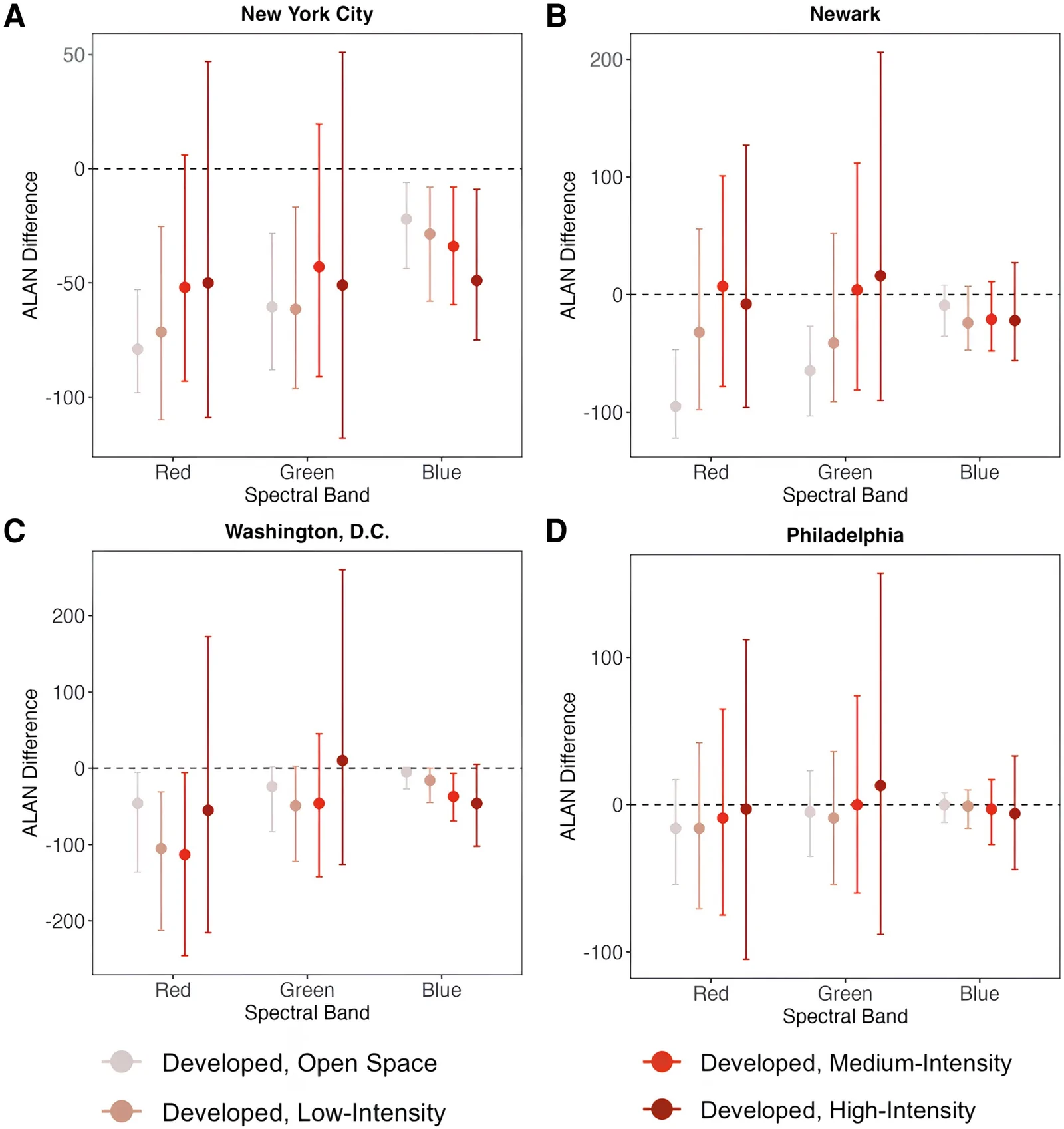
ALAN differences across urban development levels within each city from 2022 to 2023. Dots and error bars indicate median and interquartile range (25th–75th percentiles) of ALAN difference across each city and spectral band. Positive value represents higher ALAN in 2023. The horizontal dashed line indicates no change in ALAN (in 2023 compared with 2022). These values summarize spatial variability across pixels within each development class and are presented descriptively.

The observed temporal changes in ALAN from 2022 to 2023 varied greatly across spatial urban development levels within each city, with increasing ALAN levels as intensities of development rose (Figs. 3 and S6, Table S3). In NYC, open-space and low-intensity areas are associated with large negative differences in the red and green bands, whereas high-intensity areas are associated with small positive differences in red and green alongside negative differences in blue. In Newark, the urbanized areas (i.e. low, medium, and high intensity) are associated with positive differences in red and green, with blue near zero to slightly positive. On the other hand, open-space differences are negative across the three spectral bands. DC exhibits predominantly negative differences across the spectral bands and urban development levels, with stronger negative differences in red at low and medium intensities and the lone, sizable positive difference being the green band at high intensity. Philadelphia shows comparatively mixed and smaller differences across classes, including a positive green difference at high intensity. Considering apparent differences concentrated in the brightest urban cores, we interpret patterns as relative differences by development level rather than absolute changes in emission. The similarity between red and green bands in highly urbanized areas may reflect the common use of street lighting sources, such as high-pressure sodium and metal halide lamps, which emit light strongly in the yellow-orange and green-blue regions and often register as red and green in RGB-band satellite imagery (34). Thus, downtown areas, characterized by higher development intensity, could contain more sodium vapor lamps or similar sources, potentially contributing to the observed differences in the spectral patterns of ALAN.

### Mitigating ALAN corresponds with reducing shifts in phenology

**Figure 4:**
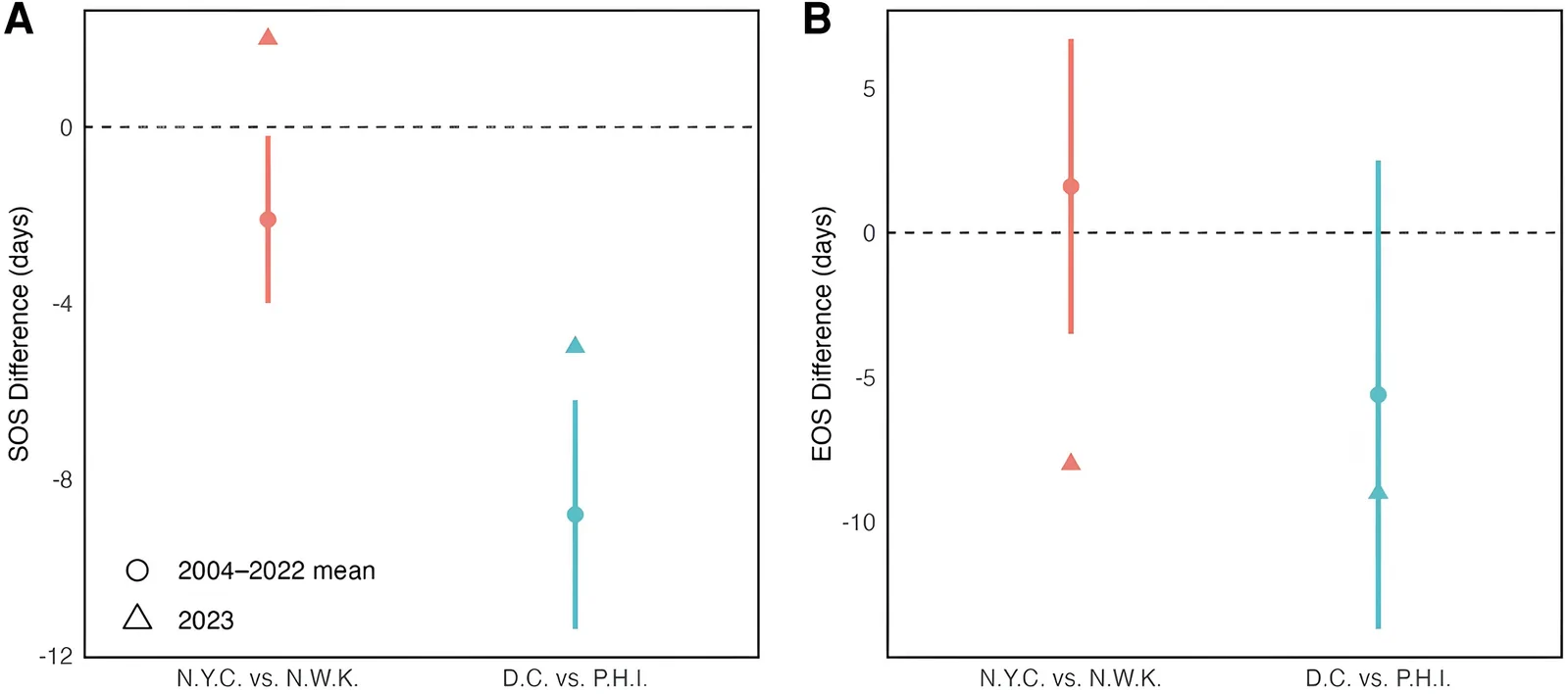
Comparison of differences in SOS (a) and EOS (b) between paired cities with and without ALAN-mitigation policies, based on long-term means (2004–2022) and postpolicy periods (2023 only). Values are calculated by subtracting phenology at ALAN policy cities from nonpolicy cities for each pair (e.g. NYC vs. Newark; DC vs. Philadelphia). Positive values indicate later phenological timing in policy cities relative to nonpolicy cities. Error bars represent 1 SD across the prepolicy years.

Cities with mitigation measures for ALAN exhibited phenological shifts that were directionally consistent with a reduced influence of artificial light. In both city pairs, nonpolicy cities initially had later SOS than policy cities (negative differences during 2004–2022), but these differences lessened or reversed after policy implementation, indicating that policy cities experienced relatively later SOS (Table S4). This pattern is consistent with a potential reduction in ALAN influence on SOS during the postpolicy period. Specifically, the SOS difference shifted from −2.1 to 2.0 days in NYC vs. Newark and from −8.8 to −5.0 days in DC vs. Philadelphia (Fig. 4a). For EOS, differences became more negative postpolicy, indicating relatively earlier senescence in the policy cities relative to the long-term mean. In NYC vs. Newark, the EOS difference shifted from 1.6 to −8.0 days, and in DC vs. Philadelphia, from −5.6 to −9.0 days (Fig. 4b), suggesting a possible correspondence between lower ALAN exposure and reduced delay in EOS in 2023. Overall, these observations are exploratory (two paired comparisons; a single postpolicy year) and should be interpreted relative to prepolicy interannual variability, motivating broader multi-city, multi-year evaluation of potential policy effects on urban phenology.

## Discussion

Our study on the potential effects of ALAN-mitigation policies suggests directional distinctions between cities with and without such policies. The variation in the levels of ALAN changes conveys the complexity of urban lighting issues and the importance of targeted interventions tailored to specific urban and spectral needs (35). Across paired comparisons, cities with ALAN-mitigation policies generally exhibited directional patterns consistent with lower ALAN from 2022 to 2023, with the contrast most evident in the blue band. Their nonpolicy neighbors, by contrast, tended to show stable or higher ALAN over the same interval. These relative differences were spatially coherent across bright urban cores and persisted when summarized at the city scale. City-level phenology summaries were directionally consistent with these lighting patterns (i.e. policy cities tending toward less advanced spring phenology and less delayed autumn phenology relative to their pairs).

The large spatial variation in ALAN temporal change across cities likely reflects differences in urban activity recovery, lighting infrastructure, and management policies. In NYC, the rebound of downtown ALAN may be linked to postpandemic increases in commercial activity, as office and business operations resumed after reductions in 2022 (36). In 2023, activity levels likely continued to return to prepandemic levels, which may have contributed to the observed increases in ALAN measurements. In contrast, DC's widespread decline in ALAN, especially in the blue band, aligns with the city's comprehensive and well-enforced lighting control initiatives (37). The city's focus on converting streetlights to fully shielded fixtures, implementing adaptive dimming, and reducing overall light output is consistent with the strong decline in blue light emission (30). Blue wavelengths are more sensitive to scattering, shielding, and dimming than red or green light, making them particularly responsive to the mitigation strategies (38). However, ALAN increased in the downtown area, particularly in the red and green bands. Philadelphia's mixed patterns likely stem from uneven progress in the Philly Streetlight Improvement Project (39), where LED retrofitting trials produced localized decreases but also potential rebound effects in other areas. Such heterogeneity underscores the complex interplay between lighting technology transitions, policy enforcement, and behavioral responses in shaping ALAN trends across urban landscapes.

Our findings suggest that policy-driven reductions in ALAN may be associated with ecological benefits, as reduced ALAN appears to lessen the advancement of SOS and the delay of EOS in urban plants. While the observed differences in SOS and EOS between paired cities are notable, the use of citywide means derived from moderate-resolution satellite data may impact the observed phenological differences due to heterogeneity in species composition, land management, and urban microclimates. Accordingly, the associations reported here should be interpreted as exploratory and hypothesis generating. Plant phenology involves a complex and multifaceted ecological process and is influenced by many environmental factors (e.g. temperature, light, and precipitation) (40). Without policies to mitigate ALAN, the continued extension of the growing season could have far-reaching consequences for ecological cycles. For example, a longer growing season could lead to increased evapotranspiration, potentially altering local water availability and hydrological processes (41). Similarly, extended plant activity might enhance carbon sequestration through prolonged photosynthesis, affecting carbon storage and microclimate conditions (42). Our proof of concept analysis highlights the value of integrating light pollution data into urban ecological research to anticipate such potential consequences. Further research across broader temporal and spatial scales is necessary to fully understand the long-term implications of ALAN on plant phenology and its cascading effects on ecosystem processes.

The findings may help policymakers to develop comprehensive lighting strategies in urban settings that encompass not only the general NTL levels but also the spectral composition of urban lighting. Policies should be localized and consider the specific ecological, social, and cultural contexts of each urban area (43, 44). Additionally, regulations could aim to curtail the use of disruptive blue light at night to protect plants, alongside other promising mitigation strategies such as tailored and shielded lights as well as luminous intensity reduction, which aim to reduce the affected area around a lamp where critical thresholds are exceeded (44), thus promoting lighting technologies and practices that minimize ecological disturbance while ensuring urban safety and functionality is paramount. Policy development should also involve stakeholders from various sectors, including urban planning, environmental conservation, and public health, to create holistic and sustainable urban lighting standards (45).

Inspired by regulatory frameworks like California's solar energy mandate for new homes, this study raises the possibility that similar mandates could be developed for urban lighting (46). Municipalities may require that new developments or major renovations incorporate sustainable luminaire designs and lighting strategies to reduce light pollution and minimize their ecological footprint (31, 44). This includes using tailored and shielded lights that prevent light from spilling upwards or sidewards, thereby reducing skyglow and environmental impact (31, 47). Employing low lumen lighting units and choosing warmer color temperatures can also mitigate the disruptive impacts of blue light emissions on plants, as suggested by the relative differences observed in this study. However, the effectiveness of spectral tuning is being debated for other taxonomic groups, such as insects and freshwater organisms (48, 49). By implementing timers and dimmers, cities can further enhance the effectiveness of their lighting, ensuring lights are only as bright as necessary and only when needed. Platforms, such as the DarkSky Approved program, offer third-party certification for products, designs, and finished projects that limit glare, prevent light trespass, and avoid night sky pollution (50). Such mandates would help not only in reducing ALAN but also in promoting energy efficiency and reducing greenhouse gas emissions, contributing to broader environmental and public health goals (5).

Our findings demonstrate the feasibility of using SDGSAT-1 to identify relative differences in ALAN levels and composition, but several limitations constrain precision and inference (51). Systematic uncertainties in NTL observations (e.g. atmospheric conditions and sensor calibration) and scene-level effects likely contribute to the observed differences even when cities are paired within the same acquisition. Percent-change metrics can inflate where baselines are small; band-specific responses (e.g. larger blue band changes) may reflect sensor spectral response, atmosphere, or illumination geometry as well as emissions; and small registration and footprint mismatches can bias pixel-wise comparisons. The short temporal scope of SDGSAT-1 further limits our ability to detect longer-term ALAN trends and phenological shifts, which limits the replication and temporal coverage of this proof of concept. Stronger inferences will require validation across additional cities and multiple postpolicy years. Additionally, the earlier overpass time (∼21:00 EST) likely underestimates policies that act later at night (e.g. Int 0274-2018 in NYC; Table 1). While VIIRS analyses provide corroborating, directional patterns, these datasets also face temporal and sensor differences that preclude definitive causal estimation in urban settings (5). Accordingly, we interpret our results as indicative rather than conclusive and recommend future work that (i) uses scene-internal pseudo-controls (e.g. reference areas within the same images) to empirically bound systematic variability, (ii) assembles longer multi-date sequences to separate measurement noise from persistent trends, (iii) undertakes cross-sensor replication to test band-specific behavior, (iv) expands paired comparisons across additional climate regions to assess generality, and (v) develops city scale, hourly observational networks to capture policy effects across the full nocturnal period.

**Table 1:** Summary of mitigation policies for study cities.

City	Policy/project	Key features/details
New York City	Int 0274-2018 (enacted on 2022 January 15)	Reduces nighttime illumination during peak bird migration periods (Aug 15–Nov 1 and Apr 1–May 31). Requires nonessential outdoor lighting in city-owned or leased buildings to be turned off between 11:00 PM and 6:00 AM during migration periods. Efforts to include similar provisions in leases where the city is not the only tenant (52).
	Int 0271-2018 (enacted on 2021 December 31)	Mandates the installation of occupancy sensors in city-owned buildings (25% by 2023, 40% by 2025, 75% by 2027, and all buildings by 2030). Requires periodic reporting on compliance (53).
	Smart Street Lighting NY Project (guidelines approved on 2016 October 13)	Offers municipalities the opportunity to convert streetlight systems to energy-efficient LED technology. Aims for cost savings, improved lighting quality, reduced energy consumption, and a smaller environmental footprint (54).
Washington DC	Site Lighting (adopted from the 2017 Green Construction Code)	Limits uplight, light trespass, and glare for all exterior lighting equipment in new constructions and level 3 alterations. Establishes exterior lighting zones with specific requirements. Includes exemptions for certain types of lighting, ensuring flexibility for critical and specialized lighting needs. Part of the broader Green Construction Code aimed at sustainable and environmentally responsible building practices (55).
	DC Smart Street Lighting Project (Detailed Scope and Benefits from RFP Documents released in early January 2021)	Convert 75,000 streetlights to energy-efficient LEDs, install remote dimming/monitoring nodes, maintain assets, and extend public Wi-Fi. Expected to result in a 50% reduction in energy use, 38,000 tons of greenhouse gas emissions eliminated annually, reduced light pollution, and lifecycle cost reduction. Enhanced equity of service, relying less on resident reports for outages, and promoting preventative maintenance for improved operational efficiencies. There is also a performance regime that includes penalties for failing to meet service and maintenance standards (56).

Taken together, our findings mark an important step toward demonstrating the feasibility of using SDGSAT-1 for urban ecological research, drawing attention to key methodological considerations such as intra-satellite image pairing, and highlighting the need for continued investigation as the dataset matures. As a result, our analysis underscores the potential, rather than the certainty, of detecting policy-related patterns in urban nighttime lighting. By effectively managing ALAN, cities may enhance the resilience and functionality of their ecosystems (57, 58). This study thus emphasizes the value of SDGSAT-1 in providing new insights into the full spectrum of light pollution impacts. It also provides a pivotal foundation for ongoing research and policy formulation, aimed at safeguarding our urban ecosystems against the unintended consequences of urbanization. As we move forward, it is imperative that we continue to refine our strategies and regulations to create more livable, sustainable, and environmentally friendly urban nightscapes.

## Materials and methods

### Data description

#### SDGSAT-1 ALAN dataset

We used ALAN images from the SDGSAT-1 satellite sensor, which possesses a panchromatic band resolution of 10 m and a multispectral (RGB) resolution of 40 m (32). SDGSAT-1's primary goal is to monitor urban NTL emissions and support sustainable development efforts, particularly related to the United Nations’ Sustainable Development Goals. Its high-resolution and spectral data, including red, green, and blue bands, offer unprecedented opportunities for studying the potential influence of ALAN-mitigation measures on ALAN spectral composition and urban environments, aiding in the formulation of targeted policies for environmental conservation and urban planning. The SDGSAT-1 satellite captures NTL in multiple spectral bands with the following wavelength ranges: blue (424–536 nm), green (506–612 nm), red (600–894 nm), and panchromatic (444–910 nm) (59). Notably, the blue band extends further into the blue region of the spectrum than the panchromatic band, enabling better sensitivity to blue-rich light sources such as LEDs. SDGSAT-1 satellite images have demonstrated remarkable efficacy in revealing spatial variations in NTL intensity, outperforming existing datasets such as the VIIRS-Day Night Band and Luojia1-1 in terms of spectral and spatial resolution (60). This capability is crucial for our study, as the change in spectral composition of ALAN can vary based on ALAN-mitigation measures and thus have varied impacts on urban tree phenology.

Each pair of cities analyzed appears within the same SDGSAT-1 image, ensuring consistency in acquisition conditions and enabling direct comparison. Furthermore, our analysis focused on relative differences between paired cities across time, rather than absolute values, to control for potential temporal variability in satellite measurements. These efforts facilitated a more accurate assessment of light pollution levels and their fluctuations over time, especially before and after the implementation of ALAN-mitigation policies by municipalities.

We used the following criteria to select images: (i) no cloud cover; (ii) spatial accuracy verified with correct georeferencing; (iii) the full city boundary of each city was included in the satellite image; (iv) to minimize seasonal variability in ALAN data, satellite images from winter (November through early February) and primary growing season (May through September) were avoided. In winter, snow cover can distort ALAN readings through higher surface albedo, while the shorter nighttime hours in summer substantially reduce the time window for NTL measurements (61). Additionally, leaf-out and leaf-fall transition periods affect ALAN observation due to dynamic leaf coverage. As a result, the dates of the images selected for the comparative analysis between NYC and Newark were 2022 March 15 (02:01 UTC) and 2023 April 20 (02:05 UTC), while the dates for DC and Philadelphia were 2022 February 21 (02:26 UTC) and 2023 October 4 (02:15 UTC).

To further validate the results and ensure the robustness of the findings, a sensitivity test was conducted by replacing one of the SDGSAT images (2023 April 20) in the pair used for the comparative analysis between NYC and Newark with an image from 2023 April 14 (02:13 UTC). We found results were largely consistent, although the magnitude of changes varied (Figs. S2 and S3). To further assess the temporal consistency of observed ALAN changes, we incorporated a newly released 2024 SDGSAT-1 satellite image for each city pair (2024 September 3 [01:59 UTC] for NYC vs. Newark; 2024 August 23 [02:01 UTC] for DC vs. Philadelphia). We repeated the comparative analysis using these later images in place of the original 2023 acquisitions, with the goal of determining whether similar directional trends in ALAN change were detectable across a longer time span. Additionally, we conducted another sensitivity test for DC and Philadelphia using the VIIRS moonlight- and atmosphere-corrected NTL product (VNP46A2) (62). The VIIRS VNP46A2 dataset is part of NASA's Black Marble product suite, providing global daily data on NTL emissions at a resolution of 500 m, represented in broadband radiance (nW/cm^2^/sr). The dataset's cloud-free, radiance-calibrated, moonlight-corrected, and atmospheric-corrected data supported the assessment of the effects of mitigation policies (62, 63).

However, these steps only improve comparability but do not eliminate all sources of uncertainty, as NTL observations are subject to atmospheric, lunar, and sensor-specific variability. Such factors can introduce inconsistencies in the radiance values both temporally and spatially. Therefore, instead of relying on the absolute temporal changes in radiance values for each city, we primarily focused on comparing spatially directional changes between paired cities over years to mitigate the influence of these systematic biases. This approach emphasizes the relative patterns of ALAN, which are generally more robust to sensor related and environmental noise. Despite this, the results from this data product should be interpreted as indicative patterns rather than definitive measurements.

#### VIIRS ALAN datasets

Because SDGSAT-1 does not provide continuous monthly data, we conducted a supplementary analysis using long-term monthly VIIRS data to evaluate broader trends beyond the temporal scope of SDGSAT-1. This VIIRS analysis serves two purposes: (i) it validates the pattern observed in SDGSAT-1 data against longer-term trends, and (ii) it provides context on ALAN trends in both cities prior to and during the SDGSAT-1 observation period, using a consistent monthly time series. This analysis strengthens our interpretation by linking short-term changes observed in SDGSAT-1 with a longer-term trend captured by VIIRS. We used the VIIRS VNP46A2 dataset and calculated the monthly mean ALAN values (in nW/cm^2^/sr) for DC and Philadelphia (2019 to 2023). Buffer zones were created at distances of 1, 11, 21, 41, and 81 km from the pixel containing the highest ALAN value in each city to capture spatial variations in ALAN intensity as one moves from the urban core to surrounding rural areas (64). We fitted linear regression models for each buffer zone in each city during the 5 years. Given DC's sustained lighting reduction policies, we hypothesize a continued decline in ALAN over time, in contrast to stable or increasing ALAN levels in Philadelphia, where no such policy existed.

#### Urban datasets

To understand ALAN distribution across varying urban development levels, we utilized the 30-m resolution National Land Cover Database (NLCD) 2021 release (65). The NLCD provides comprehensive land cover and land use information across the United States based on their landscape and percentage of impervious surface area (ISA). We focused on four urban development classes: open space (mostly vegetation in the form of lawn grasses with ISA < 20%, such as large-lot single-family homes and green spaces), low intensity (a mixture of constructed materials and vegetation with 20% < ISA < 49%, such as standard single-family homes), medium intensity (a mixture of constructed materials and vegetation with 50% < ISA < 79%, such as multi-family homes), and high intensity (mostly constructed materials with ISA > 80%, such as commercial and industrial buildings; Fig. S1). When calculating the city mean value, we only considered pixels under these four NLCD classes in our analysis and masked out all other pixels classified as other NLCD classes (e.g. open water, barren land, mixed forest, and cultivated crops).

Since the mitigation policies are implemented within the administrative urban boundary of the municipality, our study utilized the same urban boundary obtained from authoritative open data platforms, including NYC Open Data for NYC (66), City of Newark Open Data for Newark (67), Open Data PHL maps for Philadelphia (68), and Open Data DC for DC (69). We conducted our ALAN and phenology analysis using these boundaries to explore whether changes in light pollution levels and plant phenology may be associated with mitigation measures implemented within these jurisdictions. Additionally, by focusing on officially recognized urban boundaries, we ensured the reliability and relevance of our findings to policymakers and urban planners engaged in efforts to mitigate ALAN's ecological impacts.

#### Phenology dataset

To investigate the impacts of ALAN on urban tree phenology, our study utilized NASA's MODIS Land Cover Dynamics product (MCD12Q2.061) for 2022–2023, which provides yearly global data on key phenological events at a 500-m resolution (70). This dataset is widely used in ecological and climate studies due to its reliability in capturing temporal vegetation changes over large spatial scales. We used the “Greenup_1” band to represent SOS, which marks the onset of substantial vegetation greening, and the “Senescence_1” band for EOS, signaling the period when vegetation begins to senesce (71). To contextualize the observed differences in SOS and EOS between paired cities, we analyzed historical MODIS phenology data from 2004 to 2023. We extracted citywide mean SOS and EOS values for each year and calculated the annual difference between paired cities (e.g. NYC vs. Newark; DC vs. Philadelphia). We then compared the long-term mean differences (2004–2022) with those observed in the postpolicy period (2023) to evaluate whether the implementation of ALAN-mitigation measures corresponded with shifts in intercity phenological patterns.

### Study cities

The selection of study cities for this research, namely NYC and DC, alongside Newark and Philadelphia for comparison, was guided by several key considerations. Firstly, NYC and DC have implemented specific ALAN-mitigation policies, while Newark and Philadelphia, with geographical proximity and inclusion in the same SDGSAT-1 images, do not currently have any measures in place. Each pair of cities was analyzed using the same two satellite images to reduce potential systematic errors with the SDGSAT-1 data across the comparison. Secondly, all four cities are located in the Northeastern United States and experience similar climatic conditions and environmental factors. As a result, confounding variables such as variations in climate or vegetation types that could affect the study outcomes are minimized.

### Mitigation policies

NYC has implemented several notable mitigation policies aimed at reducing light pollution and improving energy efficiency in outdoor lighting. One such policy, known as Int 0274-2018, was enacted on 2022 January 15 and focuses on reducing nighttime illumination during peak bird migration periods and minimizing unnecessary lighting in city-owned spaces (52). This law requires nonessential outdoor lighting in city-owned or leased buildings where the city is the sole tenant to be turned off between 11:00 PM and 6:00 AM during migration periods, which occur from August 15 to November 1 and from April 1 to May 31. Efforts have also been made to include similar provisions in leases where the city is not the only tenant. Although the SDGSAT-1 overpass time (∼21:00 EST) precedes the curfew window and therefore cannot capture curfew-specific changes, the broader lighting reduction measures may still influence overall ALAN levels during the earlier evening period and be detectable in the SDGSAT-1 observations.

Another significant law, Int 0271-2018, was enacted on 2021 December 31 and mandates the installation of occupancy sensors to limit illumination in at least 25% of city-owned buildings by 2023, increasing to 40% by 2025, 75% by 2027, and all buildings by 2030 (53). This law also requires periodic reporting on compliance, ensuring that the goals of reduced light pollution and improved energy efficiency are being met. In addition to these city-level initiatives, the State of New York has launched the Smart Street Lighting NY project, offering municipalities the opportunity to convert their streetlight systems to energy-efficient LED technology (54). This project aims to provide cost savings, improved lighting quality, reduced energy consumption, and a smaller environmental footprint. The project was initiated following the approval of guidelines by the Public Service Commission on 2016 October 13 allowing municipalities to purchase their streetlights from the electric utility.

In DC, the DC Smart Street Lighting project represents a significant step toward reducing light pollution and improving energy efficiency in outdoor lighting (56). The project, which began in the spring of 2022, focuses on the conversion of 75,000 streetlights owned by the District Department of Transportation to energy-efficient LED technology. Each streetlight is equipped with nodes that allow for remote dimming and monitoring, enhancing the efficiency and effectiveness of streetlight operations. Lastly, project documents have also shared that all conversions are limited to warmer LED color temperatures of 2,700 or 3,000 K, which is essential in limiting blue light emissions.

The DC Smart Street Lighting project is structured as a public–private partnership (P3), with the private partner responsible for designing, building, operating, financing, and maintaining the project. The District of Columbia will compensate the private partner through milestone and availability payments based on the project's performance, ensuring that the project remains financially viable. The benefits of the DC Smart Street Lighting project are numerous. In addition to improved efficiency and effectiveness of streetlight operations, the project aims to ensure that streetlights are maintained in a state of good repair. Other benefits include equity of service, reduced reliance on resident reports for outages, at least a 50% reduction in energy use, environmental benefits such as the elimination of 38,000 tons of greenhouse gas emissions annually, reduced light pollution, and lower lifecycle costs.

### ALAN calculation

To quantify total changes in ALAN brightness, an equation was employed to combine the contributions of the red, green, and blue spectral bands (60, 72).


(1) Brightness=0.2989×R+0.5870×G+0.1140×B,

where *R*, *G*, and *B* represent the digital number (DN) value for the red, green, and blue bands, respectively. This formula is widely used in colorimetry to calculate luminance, which represents the perceived brightness of a color. We chose to calculate brightness in this way, instead of using the panchromatic band, because the panchromatic band does not include the full range of the blue band spectrum (59).

In this study, we primarily used ALAN in DN value for the analysis, following the standard approach employed in prior research (73). This practice allows for consistency with established methodologies without necessitating explicit physical parameter estimation for radiance. Previous studies revealed a strong linear correlation between simulated at-sensor radiance and DN values, indicating that DN can serve as a reliable proxy for radiance in ALAN analysis (74). However, to ensure the robustness of our findings, we further conducted a sensitivity test by converting DN values to spectral radiance (nW/cm^2^/sr/μm), as follows.

To calculate radiance (*L*) for each band in each image, we utilized the gain and bias correction given for each satellite image.


(2) L=DN×G+B,

where DN is the digital number value, the gain value is *G*, and the bias value is *B*. The gain and bias are radiometric calibration coefficients included with each SDGSAT-1 image that translate raw DNs into spectral radiance values, based on the specific response characteristics of the sensor (59).

### Statistical analysis

We calculated ALAN change by subtracting 2022 ALAN values from 2023 ALAN values of each pixel for both overall and for each spectral band:


(3) $$\Delta \mathrm{ALAN}=\mathrm{ALA}N_{2023}\text{–}\mathrm{ALA}N_{2022},$$

where ΔALAN represents the change in ALAN for a given pixel, and ALAN_2023_ and ALAN_2022_ refer to the DN values in 2023 and 2022, respectively, for each spectral band (red, green, and blue).

To further quantitatively describe the differences in ALAN between years, we calculated the percent change in total ALAN, as well as for each spectral band, by dividing the relative change (2023–2022) by 2022 value and converting it to a percentage:


(4) $$\Delta \mathrm{ALAN}\text{\%}=(\Delta \mathrm{ALAN}/\mathrm{ALA}N_{2022})\times 100\text{\%},$$

where ΔALAN% represents the percent change in ALAN, ΔALAN refers to the absolute difference in DN between 2022 and 2023, and ALAN_2022_ is the baseline DN value.

This metric provides a normalized measure of change, making it possible to compare the relative patterns of ALAN reduction between pairs of cities. To evaluate the statistical significance of the observed ALAN differences between each pair of cities, we employed a two-sample t-test (two-tailed test). To estimate the uncertainty in Fig. 2, we derived the SE for each spectral band (red, green, and blue) by dividing the SD of the difference in ALAN levels between 2022 and 2023 by the square root of the sample size for each city (count of analyzed pixels):


(5) $$\mathrm{SEM}=\sigma /\sqrt{n},$$

where SEM represents the SE of the mean, *σ* refers to the SD of ALAN change values across valid pixels, and *n* is the number of valid pixels analyzed.

These values reflect spatial variability across all analyzed pixels within each city, not temporal variability across dates, and thus provide a measure of confidence in citywide mean estimates. Then, to derive the SE for the total change in ALAN levels, we calculated the combined SE for each city by inserting the appropriate values into the ALAN Brightness equation 1 for each spectral band. These calculations describe spatial variability within single-image pairs and should not be interpreted as precise temporal trends, as systematic variation among acquisition dates may potentially occur.

We also examined the spatial distribution of these spectral changes within each city between 2022 and 2023. Since the spatial analysis used linear change, brighter areas may tend to show larger absolute changes regardless of relative variation and should be interpreted accordingly. To understand the distribution of ALAN change across different levels of urban development, we calculated the median ALAN difference values (2023–2022) for each of the three spectral bands in each city for four urban development classifications. These values reflect median differences across urban pixels and do not fully account for potential atmospheric variability or other observational uncertainties. Accordingly, results should be viewed as indicative of relative differences rather than definitive measurements.

Lastly, we assessed temporal shifts in plant phenology in relation to the timing of ALAN-mitigation policies by comparing the SOS and EOS between city pairs for long-term mean vs. postpolicy period. For each year, we then computed the difference in phenological timing between cities with and without ALAN mitigation (e.g. NYC vs. Newark; DC vs. Philadelphia). To evaluate potential policy-related shifts, we compared the long-term average differences (2004–2023) to the postpolicy period (2023) and calculated the corresponding means and SDs. 2023 was treated as a single postpolicy observation interpreted relative to prepolicy interannual variability. Considering temperature exhibits a strong influence on the phenology of urban plants, we aimed to control the temperature effect by only comparing the relative difference in SOS and EOS between geographically proximate city pairs. While other sources of variation may exist between cities, the ALAN-mitigation policy represents the most identifiable and relevant distinction likely to affect NTL levels during the study period. The selection of geographically proximate city pairs helps control for regional environmental variation. As a result, observed differences in phenological change between each city pair in postpolicy period compared with long-term mean should be interpreted as exploratory associations rather than causal relationships, as contributions from other factors cannot be ruled out and merit further investigation through field-based studies.

## Supplementary Material

pgag237_Supplementary_Data

## Data Availability

All the data necessary to evaluate the results in this paper are present in the paper and/or Supplementary material. All the codes are available on Open Science Framework at https://osf.io/wkpxj/.
