# Supplementary material for: Mitigating artificial light at night: Effectiveness of policies and implications for plant phenology
Source: PNAS Nexus. 2026 Aug 4;5(8):pgag237. doi: 10.1093/pnasnexus/pgag237 (PMC13435329; doi:10.1093/pnasnexus/pgag237)
Supplement: Supplementary file 1 [file pgag237_supplementary_data.pdf]

Supplementary Materials

Mitigating artificial light at night: effectiveness of policies and implications for plant phenology

Brandt Geist[1], Lin Meng[1*], Travis Longcore[2], Zhuosen Wang[3,4], Franz Hölker[5,6], Huidong Li[1], Hao Yin[1], Mahir Tajwar[1]

[1] Department of Earth and Environmental Sciences, Vanderbilt University, Nashville, TN, 37235, USA
[2] Institute of the Environment and Sustainability, University of California, Los Angeles, Los Angeles, CA, 90095, USA
[3] Earth System Science Interdisciplinary Center, University of Maryland, College Park, MD, 20742, USA
[4] NASA Goddard Space Flight Center, Greenbelt, MD, 20771, USA
[5] Leibniz Institute of Freshwater Ecology and Inland Fisheries (IGB), Müggelseedamm 310, 12587, Berlin, Germany
[6] Institute of Biology, Freie Universität Berlin, Königin-Luise-Straße 1-3, 14195, Berlin, Germany

[*] Corresponding Author: Lin Meng (lin.meng@vanderbilt.edu)

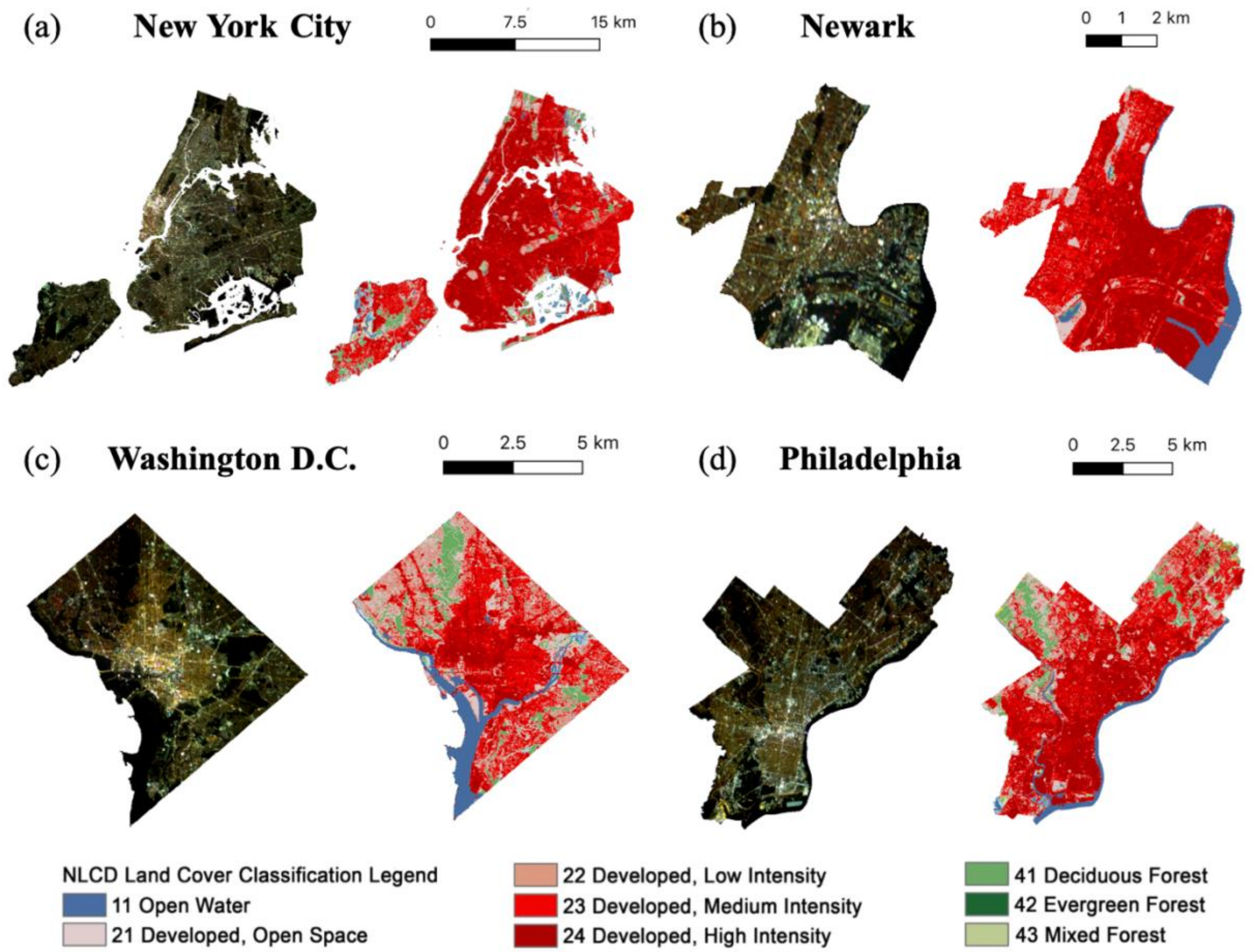

Figure S1. SDGSAT-1 Nighttime light images and land cover classifications across the four cities in 2023. On the left side of each panel are satellite nighttime light images of each city. On the right side are the corresponding land cover classifications based on the National Land Cover Database.

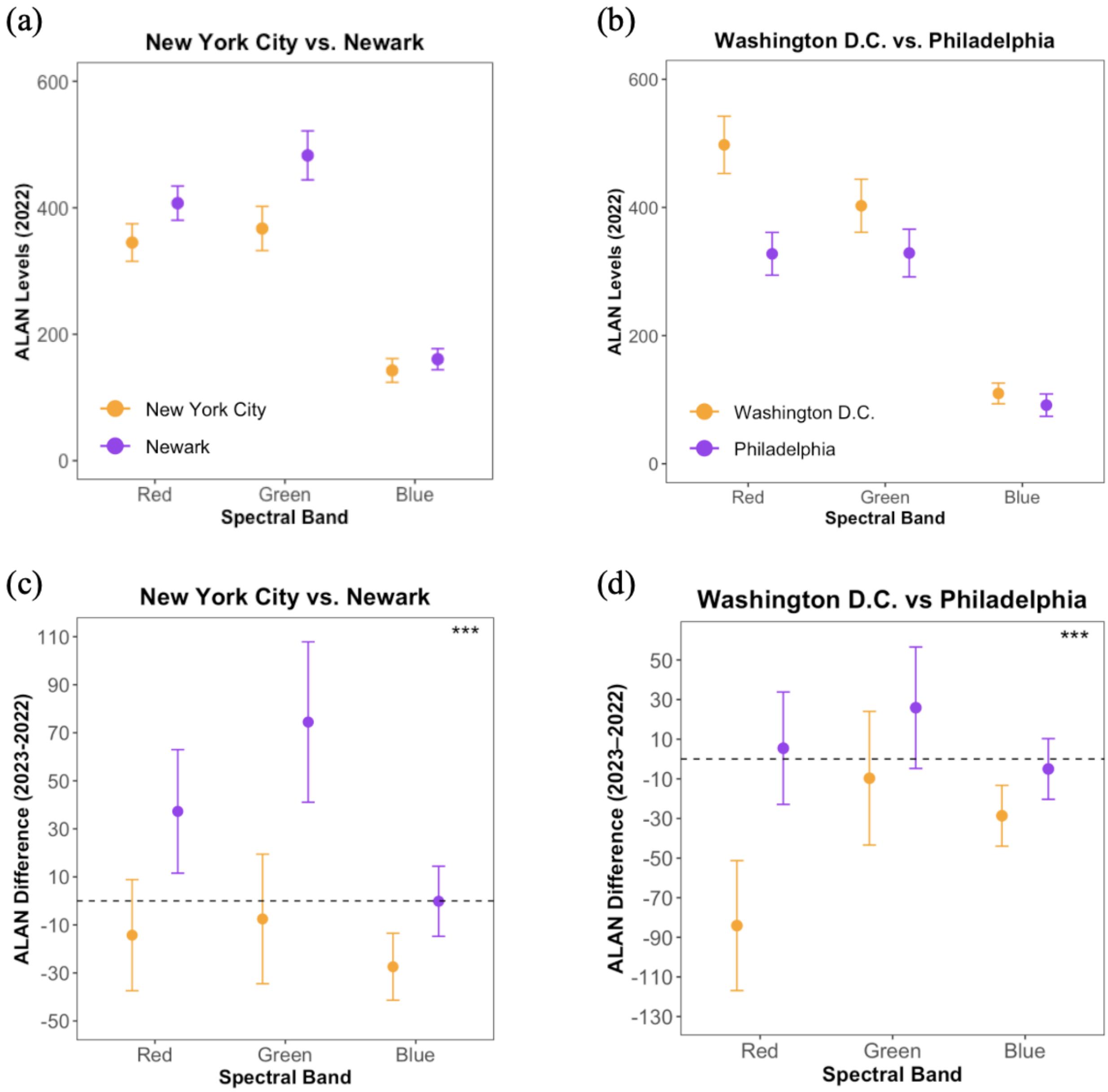

Figure S2. Reduced ALAN for cities with ALAN-mitigation policies (New York City and Washington D.C.) compared to cities without such policies (Newark and Philadelphia) for each spectral band (red, green, and blue). Dots and error bars show the mean values across each city and a 10% standard deviation. Panels a and b show ALAN levels based on mean DN values for 2022. Panels c and d show the statistically significant reductions in DN values (2023 - 2022) indicated by *** for $p < 0.001$.

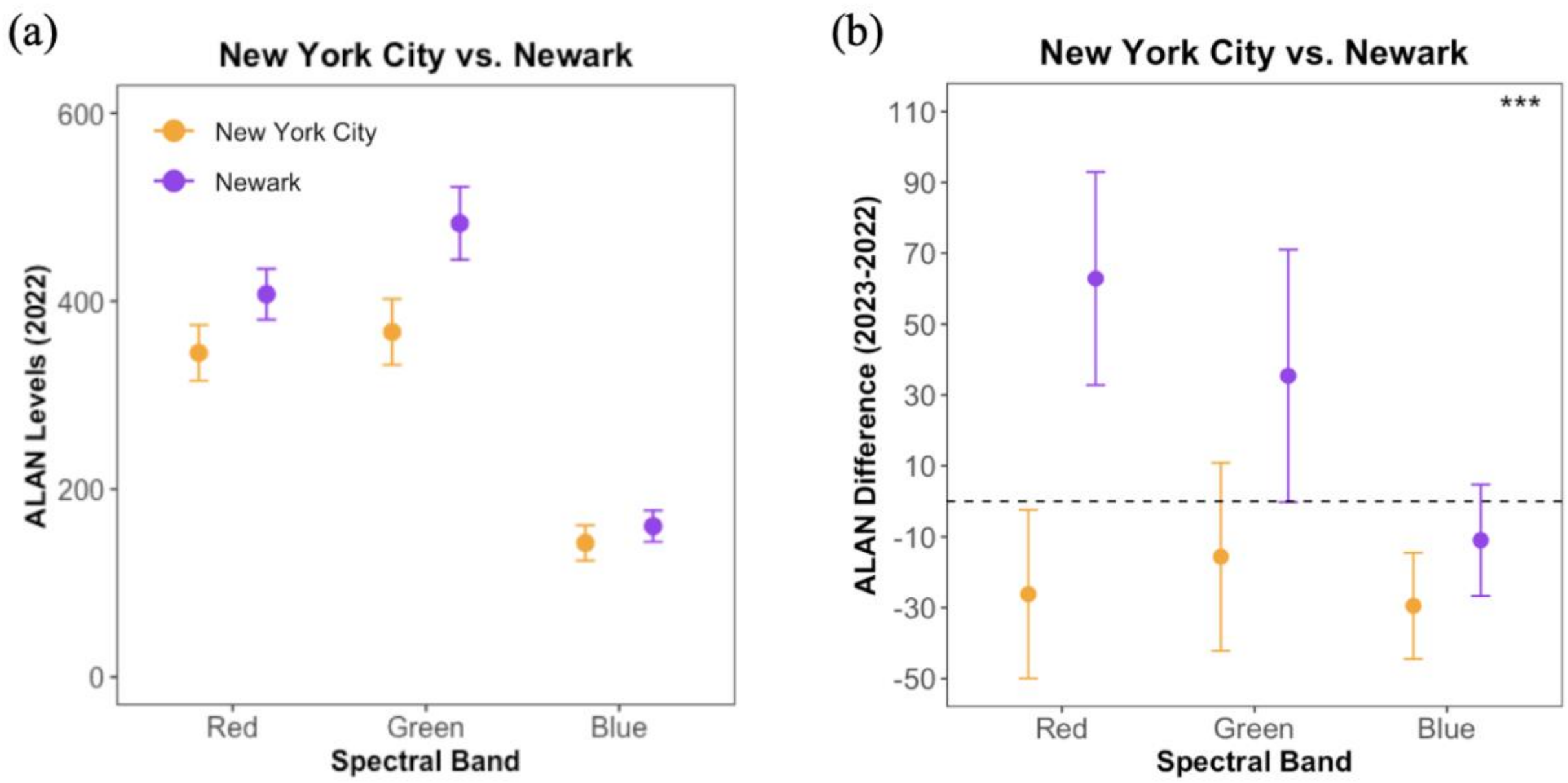

Figure S3. ALAN levels in 2022 and ALAN difference between 2022 and 2023 for each spectral band (red, green, and blue) between New York City and Newark. As a sensitivity test, the original satellite image for 2023 (April 20, 2023, Fig. S2), was replaced with a satellite image from April 14, 2023. The figure aims to demonstrate if substituting one of the satellite images yields similar ALAN change patterns and supports the main findings. (a) displays the ALAN baseline for each spectral band (red, green, and blue). (b) illustrates the mean differences in ALAN values, with statistically significant reductions indicated by *** for $p < 0.001$. Error bars represent a 10% standard deviation.

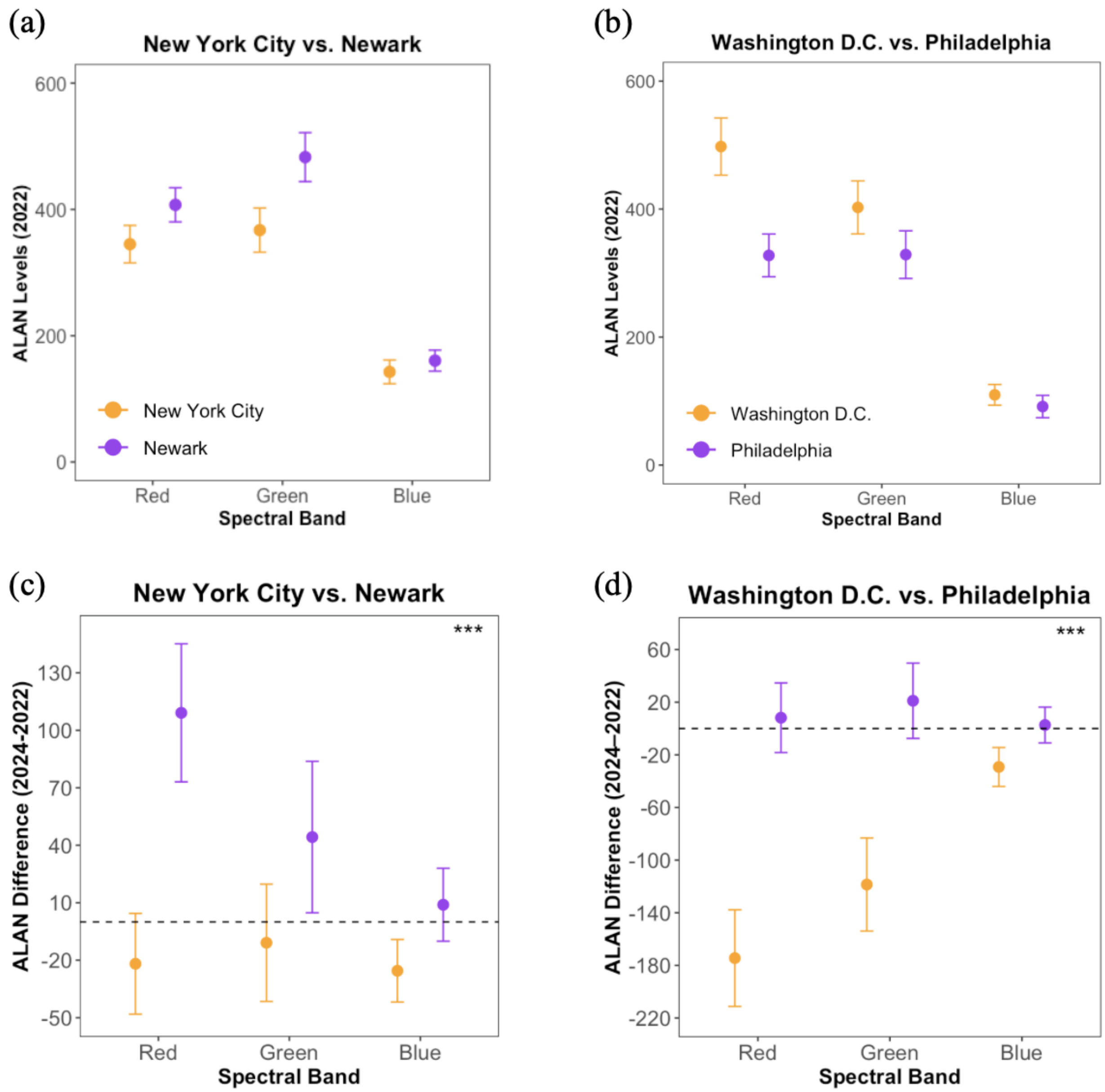

Figure S4. ALAN levels in 2022 and ALAN difference between 2022 and 2024 for each spectral band (red, green, and blue) of New York City vs. Newark and Washington D.C. vs. Philadelphia. As a sensitivity test, the respective satellite image for 2023 of each city pair was replaced in the comparative analysis with a satellite image from 2024 (September 3 for New York City vs. Newark; August 23 for Washington D.C. vs. Philadelphia). The figure aims to demonstrate if substituting one of the satellite images yields similar ALAN change patterns across greater temporal scales and supports the main findings. (a and b) displays the ALAN baseline for each spectral band (red, green, and blue). (c and d) illustrates the mean differences in ALAN values, with statistically significant reductions indicated by *** for $p < 0.001$. Error bars represent a 10% standard deviation.

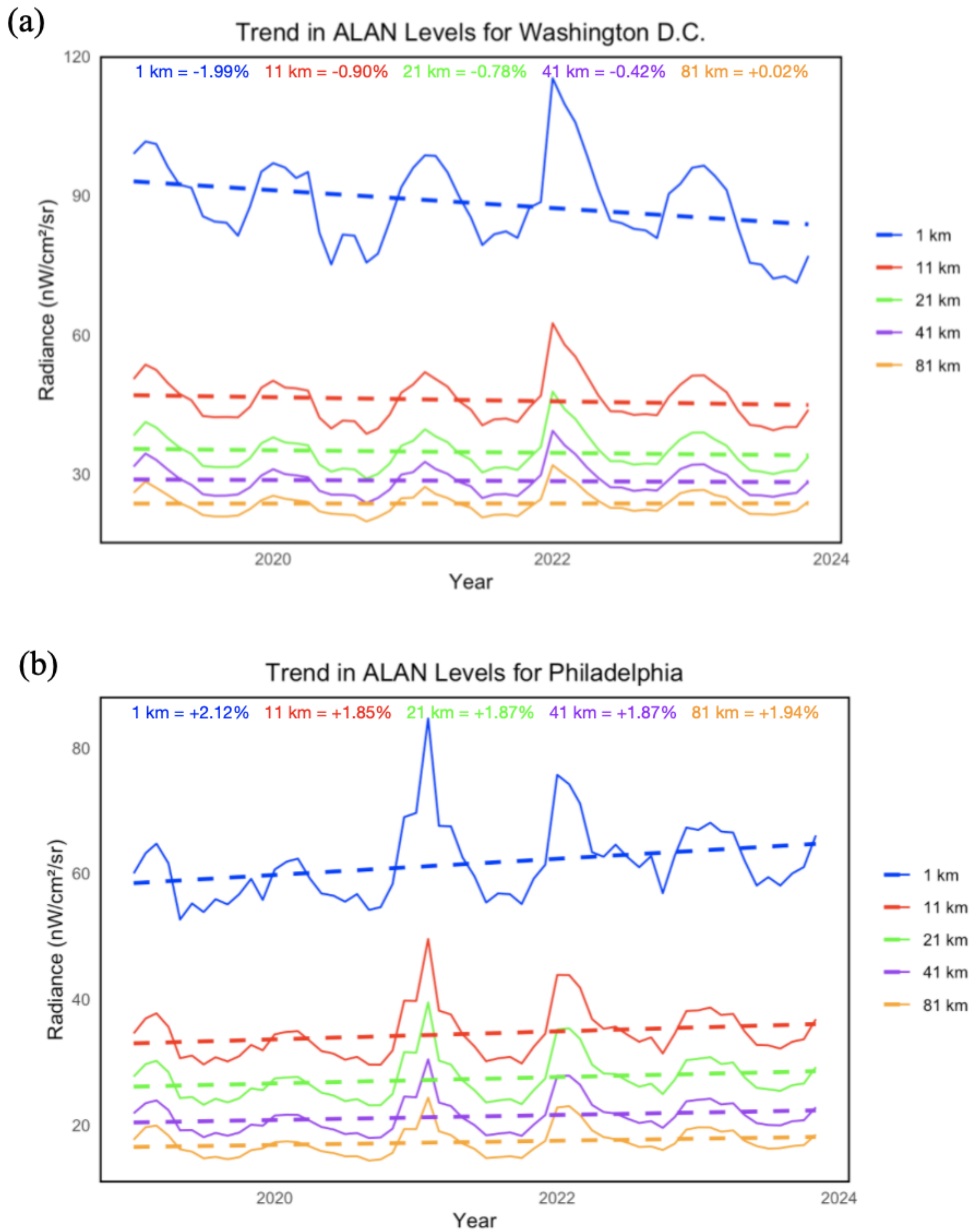

Figure S5. Monthly ALAN for Washington D.C. (a) and Philadelphia (b) using the VIIRS dataset. Broadband radiance of ALAN (nW/cm^2/sr) was shown at varying buffer distances (1 km, 11 km, 21 km, 41 km, and 81 km) from the pixel with the highest ALAN value. Dashed lines represent linear regression fitting for each buffer distance. Annual trends are expressed as percent change per year and were calculated using 2023 value minus 2019 value, divided by the initial value of 2019, and then divided by 5 years.

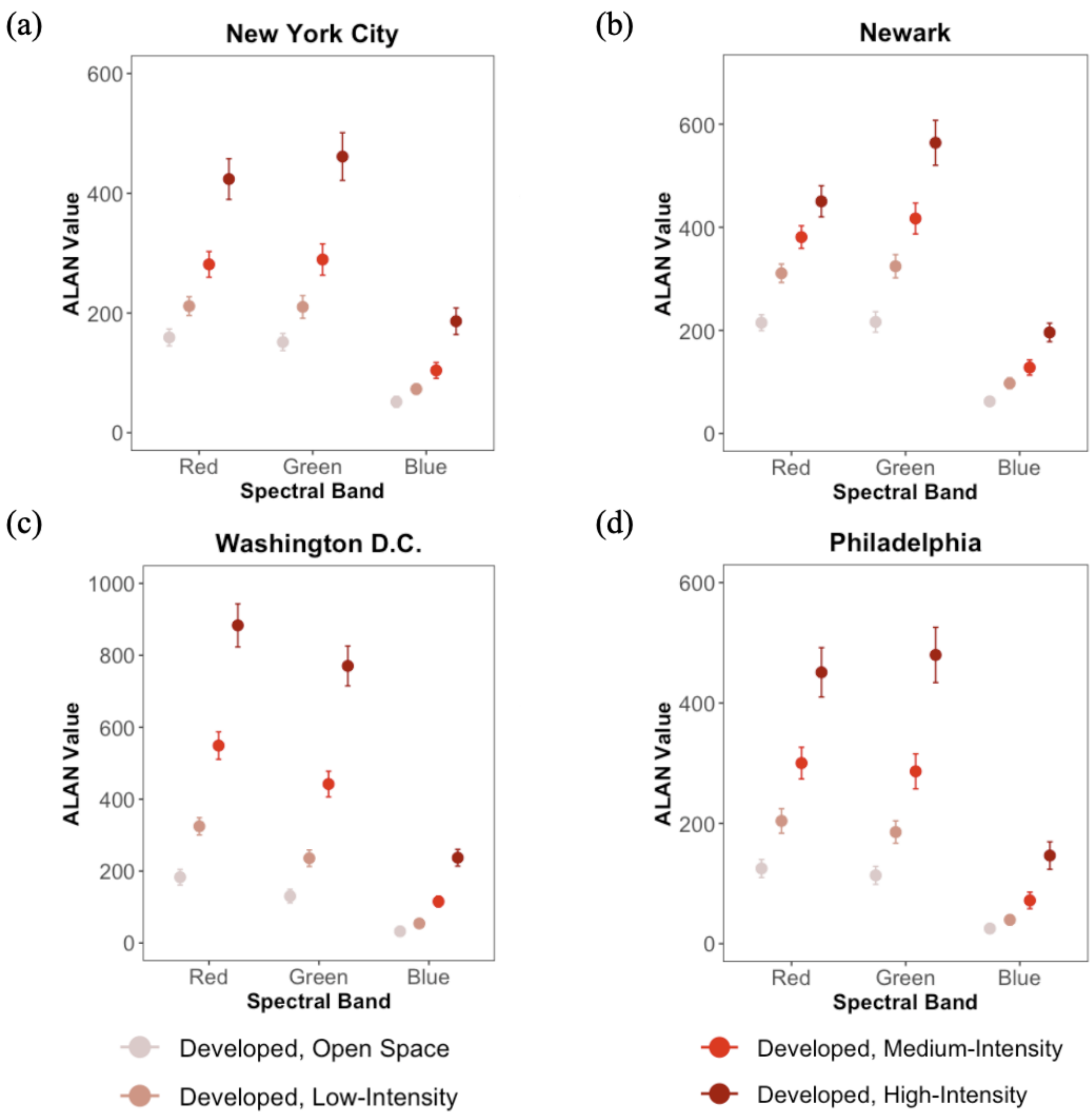

Figure S6. ALAN variations across urban development levels in 2022. Error bars represent 10% standard within each level.

Table S1. Overall and band-specific changes in nighttime light levels for paired cities with and without ALAN-mitigation policies. Values are based on change in digital number (DN) and reported as mean percent change ± SE with corresponding p-values (Equations 1, 3, 4, and 5).

City	Band	DN Change	Percent Change	p-value
New York City	Overall		-4.63 ± 1.20%	< 0.001
	Red	-14.28	-4.14 ± 1.13%	< 0.001
	Green	-7.52	-2.05 ± 1.33%	< 0.001
	Blue	-27.42	-19.22 ± 0.71%	< 0.001
Newark	Overall		11.78 ± 4.12%	< 0.001
	Red	37.27	9.15 ± 3.41%	< 0.001
	Green	74.46	15.42 ± 4.88%	< 0.001
	Blue	-0.16	-0.10 ± 2.10%	< 0.001
Washington D.C.	Overall		-9.44 ± 3.30%	< 0.001
	Red	-28.63	-16.89 ± 3.73%	< 0.001
	Green	-9.70	-2.41 ± 3.46%	< 0.001
	Blue	-84.08	-26.10 ± 1.36%	< 0.001
Philadelphia	Overall		4.49 ± 1.91%	< 0.001
	Red	5.45	1.66 ± 1.88%	< 0.001
	Green	25.90	7.88 ± 2.10%	< 0.001
	Blue	-5.02	-5.49 ± 0.98%	< 0.001

Table S2. ALAN in spectral radiance (x10^5 nW/cm^2/sr/μm) for red, green, and blue bands across selected dates in four cities (New York City, Newark, Washington D.C., and Philadelphia).

City	Image Date	Red Band	Green Band	Blue Band
New York City	March 15, 2022	468.47	186.78	142.60
	April 14, 2023	432.21	178.47	113.22
	April 20, 2023	447.93	182.40	114.86
Newark	March 15, 2022	552.80	245.40	160.31
	April 14, 2023	639.25	264.17	149.51
	April 20, 2023	605.25	284.21	160.36
Washington D.C.	February 21, 2022	675.26	204.73	109.88
	October 4, 2023	425.53	164.68	58.51
Philadelphia	February 21, 2022	444.90	167.34	91.75
	October 4, 2023	341.37	148.18	61.72

Table S3. Band-specific differences in nighttime light levels across urban development level within each city. Values are reported as the change in digital number (DN) and ± 10% standard deviation (SD) with corresponding p-values.

City	Development Level	Band	DN Change	p-value
New York City	Open Space	Red	-68.41 ± 11.82	> 0.05
		Green	-51.02 ± 14.28	> 0.05
		Blue	-24.62 ± 6.40	> 0.05
	Low-intensity	Red	-50.11 ± 17.32	< 0.001
		Green	-36.35 ± 20.44	< 0.001
		Blue	-28.80 ± 9.49	< 0.001
	Medium-intensity	Red	-27.08 ± 18.33	< 0.001
		Green	-15.45 ± 21.71	< 0.001
		Blue	-27.97 ± 11.02	< 0.001
	High-intensity	Red	4.64 ± 26.91	< 0.001
		Green	6.26 ± 31.29	< 0.001
		Blue	-27.14 ± 16.46	< 0.001
Newark	Open Space	Red	-43.13 ± 26.99	> 0.05
		Green	-17.91 ± 29.06	> 0.05
		Blue	3.06 ± 16.54	> 0.05
	Low-intensity	Red	21.94 ± 30.28	< 0.001
		Green	42.71 ± 34.91	< 0.001
		Blue	4.16 ± 18.11	< 0.001
	Medium-intensity	Red	36.16 ± 22.14	< 0.001
		Green	53.00 ± 28.08	< 0.001
		Blue	-8.18 ± 11.06	< 0.001
	High-intensity	Red	46.49 ± 27.05	< 0.001
		Green	99.85 ± 36.34	< 0.001
		Blue	4.31 ± 15.93	< 0.001
Washington D.C.	Open Space	Red	-77.09 ± 18.95	< 0.001
		Green	-30.98 ± 19.12	< 0.001
		Blue	-12.45 ± 8.40	< 0.001
	Low-intensity	Red	-118.87 ± 21.50	< 0.001
		Green	-49.24 ± 22.42	< 0.001
		Blue	-23.02 ± 9.90	< 0.001
	Medium-intensity	Red	-101.09 ± 35.12	< 0.001
		Green	-19.55 ± 35.25	< 0.001
		Blue	-33.83 ± 16.55	< 0.001
	High-intensity	Red	-3.67 ± 45.77	< 0.001
		Green	84.58 ± 48.02	< 0.05
		Blue	-38.35 ± 21.86	> 0.05
Philadelphia	Open Space	Red	4.55 ± 23.96	< 0.001
		Green	9.22 ± 21.69	< 0.001
		Blue	-0.92 ± 6.86	< 0.001
	Low-intensity	Red	-5.05 ± 20.59	< 0.001

		Green	-1.04 ± 18.85	< 0.001
		Blue	-4.19 ± 8.76	< 0.001
	Medium-intensity	Red	2.75 ± 24.87	< 0.001
		Green	18.19 ± 26.44	< 0.001
		Blue	-4.19 ± 12.47	< 0.001
	High-intensity	Red	11.80 ± 34.29	< 0.001
		Green	47.10 ± 38.65	< 0.05
		Blue	-7.27 ± 20.42	> 0.05

Table S4. Mean start of season (SOS) and end of season (EOS) (day of year) for each city during the long-term (2004–2022) and post-policy (2023) periods, derived from MODIS phenology data.

	New York City		Newark		Washington D.C.		Philadelphia	
	Long-Term	Post-Policy	Long-Term	Post-Policy	Long-Term	Post-Policy	Long-Term	Post-Policy
SOS	103.1	95	105.1	93	81.8	74	90.6	79
EOS	215.2	219	213.6	227	209.6	220	215.2	229
